# Epitaxy and transport properties of alkali-earth palladate thin films

**DOI:** 10.1080/14686996.2023.2265431

**Published:** 2023-10-18

**Authors:** Yusuke Kozuka, Taisuke T. Sasaki, Terumasa Tadano, Jun Fujioka

**Affiliations:** a Research Center for Materials Nanoarchitectonics, National Institute for Materials Science (NIMS), Tsukuba, Japan; b Research Center for Magnetic and Spintronic Materials, National Institute for Materials Science (NIMS), Tsukuba, Japan; c Department of Material Science, University of Tsukuba, Tsukuba, Japan

**Keywords:** Palladate, thin film, transmission electron microscopy, first-principles calculation, transport properties

## Abstract

Topological insulators and semimetals are an interesting class of materials for new electronic and optical applications owing to their characteristic electromagnetic responses originating from the spin-orbit coupled band structures. However, topological electronic structures are rare in oxide materials despite their chemical stability being preferable for applications. In this study, given the theoretical prediction of Dirac bands in CaPd_3_O_4_, we investigate the fabrication and transport properties of SrPd_3_O_4_ and CaPd_3_O_4_ thin films as candidates of oxide Dirac semimetals. We have found that these materials are epitaxially grown on MgO (100) substrate under limited growth conditions by pulsed laser deposition. The transport properties show a weak temperature dependence, suggestive of narrow-gap properties, although unintentionally doped holes hinder us from revealing the presence of the Dirac band. Our study establishes the basic thermodynamics of thin-film fabrication of these materials and will lead to interesting properties characteristic of topological band structure by modulating the electronic structure by, for example, chemical substitutions or pressure.

## IMPACT STATEMENT

We report the fabrication and transport properties of NaPt_3_O_4_-type alkali-earth palladate thin films for the first time, adding a new oxide narrow-gap material to the field of oxide electronics.

## GRAPHICAL ABSTRACT

**Figure:**
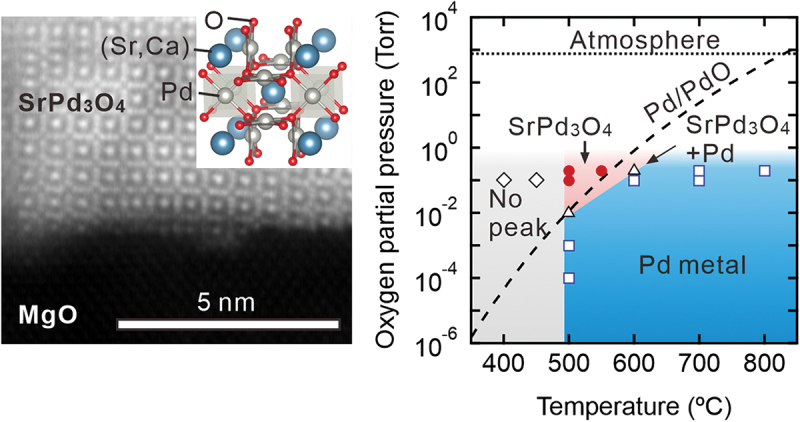

## 1. Introduction

Since the discovery of topological insulators and semimetals, a number of topological materials have been predicted and experimentally confirmed [1–5]. Topologically nontrivial band structures are realized when multiple bands with different symmetries are hybridized with each other, and therefore, narrow band-gap materials with strong spin–orbit interaction are straightforward choices. Along this guideline, pnictides and chalcogenides containing heavy elements such as tellurides, antimonides, and bismuthides are representative candidates [2–4].

Beyond these standard topological materials, transition-metal oxides are an interesting class of materials as they possess a strong correlation as well. Among them, Ir oxides including perovskite and pyrochlore materials have been intensively studied because of the competing energy scales of electron correlation and spin–orbit interaction, which leads to various topological phases [6–8] in response to environmental changes such as temperature, magnetic field, and pressure [9–12].

Utilizing thin-film techniques, the low-dimensional and interface properties have additionally been investigated, enabling the artificial design of correlated topological properties [13–15]. As one of the advantages of oxide thin films, the influences of epitaxial strain have been intensively studied for perovskite oxides owing to their flexibility on the lattice and the variety of the choice of substrates [16]. As a result, structural changes such as *M*O_6_ (*M*: transition metal) octahedral rotations or lattice compression/elongation have been utilized to drastically alter the electronic structure via the modulations of band width as well as underlying crystal symmetry, causing, for example, the carrier-type change, or mobility enhancement in zero-gap or narrow-gap oxides [17,18]. Despite such intriguing possibilities, however, the thin films of topological transition-metal oxides are rarely studied other than Iridates [17] or Niobates [18].

**Figure 1.**
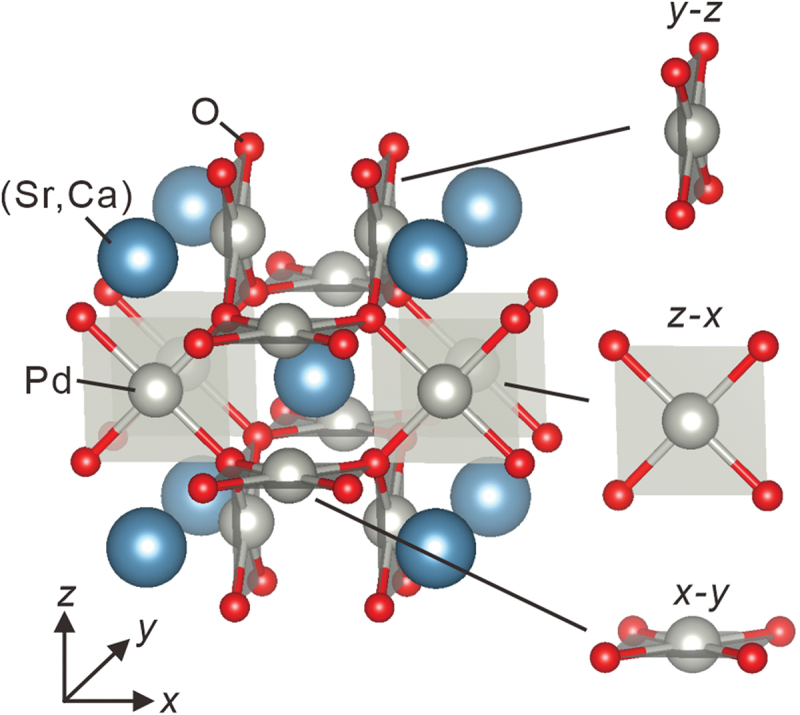
The crystal structure of SrPd_3_O_4_ and CaPd_3_O_4_ produced by VESTA [33]. Square planar-coordinated PdO_4_ plaquettes are stacked along all three crystallographic directions.

Palladium oxides are thought to be other candidates for topological materials as some of them have been predicted to involve narrow or zero band gaps by first-principles calculations [19–21]. Experimentally, the bulk properties of some of the palladates have been investigated, and zero-gap or narrow-gap properties have been revealed [22–24]. In contrast, palladate thin films have been scarcely fabricated except for a few examples such as PbPdO_2_ (Refs. [25–31]). Here, we report the fabrication of *A*Pd_3_O_4_ thin films (*A*: Ca or Sr) and their transport properties, which have not been achieved so far. This class of materials possesses a NaPt_3_O_4_-type crystal structure [32], where square planar-coordinated Pd plaquettes are stacked along all three crystallographic directions as shown in Figure 1 [33]. Unlike perovskite-type oxides, it is still not clear whether strain engineering is feasible in NaPt_3_O_4_-type materials due to the lack of studies of thin-film fabrication.

In this study, we find that *A*Pd_3_O_4_ thin films can be epitaxially grown on MgO (100) substrate under limited growth conditions by pulsed laser deposition, while a single phase is not obtained for other typical oxide substrates such as SrTiO_3_ or MgAl_2_O_4_ within our investigation. The electrical transport measurements show that CaPd_3_O_4_ thin film is weakly insulating, whereas SrPd_3_O_4_ thin film is metallic with a slight upturn at low temperatures, suggestive of proximity to band gap closing. First-principles band calculation also supports narrow-gap properties of CaPd_3_O_4_ and SrPd_3_O_4_. Our study indicates that these compounds would be promising candidates as new topological materials by appropriate chemical substitution or by external stimuli such as pressure.

## 2. Experimental details

*A*Pd_3_O_4_ thin films are grown by pulsed laser deposition using the fourth harmonic wave of the Nd:YAG (LS-2145TF, LOTIS TII, Belarus) laser with a typical laser energy of 20 mJ/pulse. The targets are made by the solid-state reaction of *A*CO_3_ and PdO powder at a molar ratio of 1:3 at 1200°C for 24 h in the air. We have employed MgO (100) (*a* = 4.21 Å) and MgAl_2_O_4_ (*a* = 8.08 Å, *a*/2 = 4.04 Å) substrates because of their bulk lattice constants close to SrPd_3_O_4_ (*a* = 5.83 Å, $a/\sqrt{2}$ = 4.12 Å) and CaPd_3_O_4_ (*a* = 5.75 Å, $a/\sqrt{2}$ = 4.06 Å) when the lattice is rotated by 45º around the out-of-plane axis. We have also tried SrTiO_3_ (*a* = 3.91 Å) and GdScO_3_ (*a* = 5.54 Å, *b* = 5.71 Å, *c* = 7.93 Å, $a_{pseudo}=\sqrt{\left(2a^{2}+2b^{2}+c^{2}\right)/12}=3.96$ Å) substrates for comparison. The crystal structure and film quality are characterized by X-ray diffraction (SmartLab, Rigaku Co., Japan) and (scanning) transmission electron microscope ((S)TEM) (Titan G2 80-200, Thermo Fisher Scientific, USA). Thin-lamella specimens for the cross-sectional (S)TEM observation were prepared using a focused ion beam (FIB) (Helios G4 UX, Thermo Fisher Scientific, USA). Transport properties are measured by a standard four-probe method using a cryostat equipped with a 14 T superconducting magnet (PPMS DynaCool, Quantum Design, USA). Electrical contacts are made by ultrasonically binding the Al wires on the surface of the samples. We have used an excitation current of 1 μA, while we have confirmed that the resistivity is not affected by the current at least between 0.1 and 100 μA.

The band structures are calculated based on density functional theory using the *Vienna ab-initio simulation package* (VASP) [34], which implements the projector augmented wave (PAW) method [35] with including the spin-orbit coupling. The plane-wave kinetic energy cutoff was set to 400 eV, and 6 × 6 × 6 Γ-centred k points were used for the Brillouin zone integration. The Heyd-Scuseria-Ernzerhof (HSE06) screened hybrid functional is used [36–38].

## 3. Thin film growth and structural and chemical characterization

**Figure 2.**
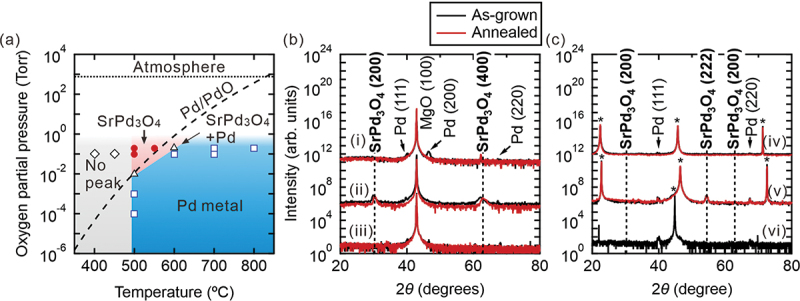
(a) Phase stability of SrPd_3_O_4_ thin film grown by pulsed laser deposition on MgO (100) substrate as functions of growth temperature (*T*_g_) and oxygen partial pressure (PO_2_). The phase equilibrium curve of Pd/PdO is also shown as a guide. (b) *θ*-2*θ* scan of X-ray diffraction of SrPd_3_O_4_ thin films under different growth conditions: (i) *T*_g_ = 700°C, P_O2_ = 0.2 Torr, (ii) *T*_g_ = 500°C, P_O2_ = 0.2 Torr, (iii) *T*_g_ = 450°C, P_O2_ = 0.1 Torr. (c) X-ray diffraction of SrPd_3_O_4_ thin films grown on different substrates at 500°C under 0.2 Torr: (iv) GdScO_3_ (110) substrate, (v) SrTiO_3_ (100) substrate, (vi) MgAl_2_O_4_ (100) substrate. Asterisks in (c) indicate the peaks of substrates. In (b) and (c), black and red curves are the data of as-grown films and the films annealed at 800°C in the air for 12 hours, respectively. The data of X-ray diffraction are shifted vertically for clarity.

The optimization of the thin-film growth of SrPd_3_O_4_ on MgO (100) substrate is summarized in Figure 2(a), where substrate temperature and oxygen partial pressure are varied as typical thermodynamic variables. Here, the phases are determined from the peak positions of X-ray diffraction as some of the typical diffraction patterns shown in Figure 2(b). At temperatures lower than 500°C, no indication of crystal phases is observed irrespective of the oxygen partial pressure, probably due to the low thermal energy for crystallization. On the other hand, the Pd metal is segregated at high temperatures above 600°C. The single phase of SrPd_3_O_4_ thin films is obtained only around 500°C above 0.2 Torr oxygen partial pressure. Small Pd metal peaks sometimes remain in the X-ray diffraction, which is diminished by annealing at 800°C in a flowing oxygen atmosphere. This phase diagram is understood based on the Ellingham diagram of the phase equilibrium curve of Pd/PdO calculated from the Gibbs free energy for bulk as indicated by the dashed curve in Figure 2(a) [39]. This tendency contrasts with the case of many oxides, where thin films are frequently stabilized at much wider growth conditions than those of the bulk when grown on an isostructural substrate as termed ‘epitaxial stabilization’ [40].

We also try growing SrPd_3_O_4_ thin films on various substrates other than MgO (100) as shown in the X-ray diffraction patterns in Figure 2(c) but find that SrPd_3_O_4_ films are not epitaxially grown on MgAl_2_O_4_ (100), SrTiO_3_ (100), GdScO_3_ (110) substrates, while small SrPd_3_O_4_ (111) peaks are seen in the case of SrTiO_3_ (100) substrate. In a sense, this is a surprising result because the lattice constant of MgAl_2_O_4_ is better matched with SrPd_3_O_4_ than that of MgO, which will be discussed later together with the results of TEM.

**Figure 3.**
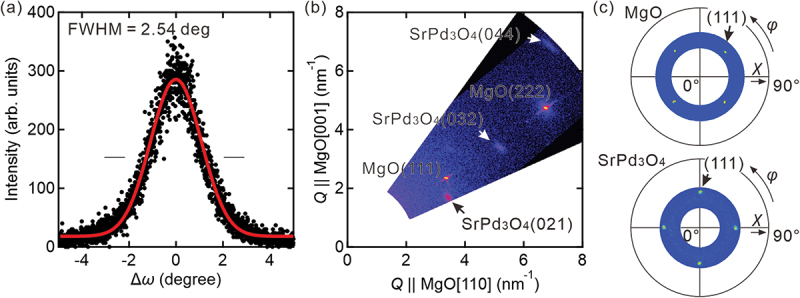
(a) Rocking curve around the SrPd_3_O_4_ (200) peak, (b) Reciprocal space mapping, and (c) Pole figure. In (c), The pole figure of the MgO substrate is also shown to clarify the in-plane relationship between the film and the substrate.

The crystal quality (mosaicity) of the SrPd_3_O_4_ film grown on MgO (100) substrate is evaluated by the full-width at half-maximum of the rocking curve around the SrPd_3_O_4_ (200) peak, which is 2.54º as shown in Figure 3(a). This value indicates a moderate crystal quality with a relatively large distribution of grain orientations. The epitaxial relationship is confirmed by reciprocal space mapping (Figure 3(b)) and pole figures (Figure 3(c)). These measurements show the [100] axis of the SrPd_3_O_4_ film is along the [110] direction of the MgO substrate for better lattice matching. The pole figure clearly shows the four-fold symmetry of the SrPd_3_O_4_ film. We have also investigated the growth of CaPd_3_O_4_ films and found almost the same tendency as SrPd_3_O_4_. The lattice constants of SrPd_3_O_4_ and CaPd_3_O_4_ thin films are extracted from the reciprocal space mapping as 5.78 Å (in-plane) and 5.91 Å (out-of-plane) for SrPd_3_O_4_ and 5.75 Å (in-plane) and 5.72 Å (out-of-plane) for CaPd_3_O_4_.

**Figure 4.**
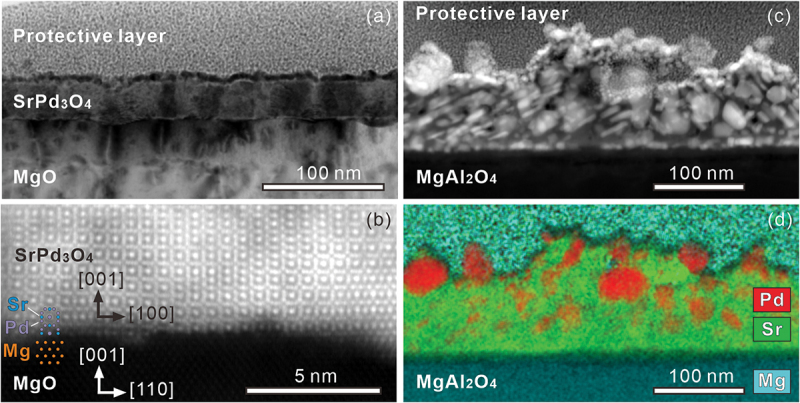
(a) A bright-field transmission electron microscope image and (b) a high-magnification HAADF-STEM image of a SrPd_3_O_4_ film grown on MgO (100) substrate. (c) A low-magnification HAADF-STEM image and (d) an EDS map of a SrPd_3_O_4_ film grown on MgAl_2_O_4_ (100) substrate.

To further obtain insight, the microstructure is analyzed by cross-sectional TEM observation. Figure 4(a) shows a bright-field TEM image of a SrPd_3_O_4_ thin film grown on a MgO (100) substrate, where columnar growth of the SrPd_3_O_4_ layer is clearly visible due to the different diffraction contrasts. A high-magnification high-angle annular dark field STEM (HAADF-STEM) image in Figure 4(b) shows, as found by X-ray diffraction, (100)-oriented epitaxial growth is confirmed. In contrast to the SrPd_3_O_4_ layer grown on the MgO substrate, a low-magnification HAADF-STEM image in Figure 4(c) indicates the occurrence of the phase separation within the SrPd_3_O_4_ layer grown on the MgAl_2_O_4_ substrate. The elemental mapping by energy-dispersive X-ray spectroscopy (EDS) in Figure 4(d) indicates that the SrPd_3_O_4_ layer phase-separates into Pd metal and SrO_*x*_.

**Figure 5.**
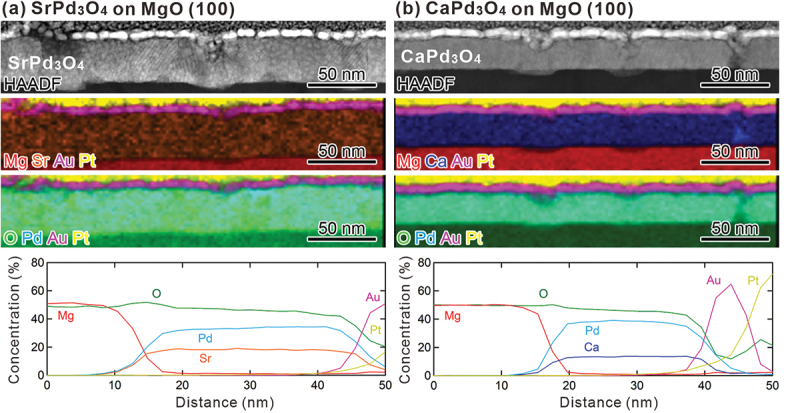
HAADF-STEM images, EDS maps, and line scans of the EDS maps for (a) SrPd_3_O_4_ and (b) CaPd_3_O_4_ films grown on MgO (100) substrates. Au and Pt layers on the films are the protective layers deposited for the TEM specimen fabrication.

The chemical compositions of SrPd_3_O_4_ and CaPd_3_O_4_ films grown on MgO (100) substrates are characterized by EDS mapping as shown in Figure 5. We find that all the elements are almost uniformly distributed within the films without significant segregation. For CaPd_3_O_4_ thin film, the cation ratio is nearly Pd/Ca ≈ 3, consistent with the designed composition, whereas Pd/Sr ≈ 2 in the case of SrPd_3_O_4_ thin film. The deviation from the designed composition of SrPd_3_O_4_ film may be due to incongruent ablation of pulsed laser between Sr and Pd.

## 4. Discussions on thin film growth

**Figure 6.**
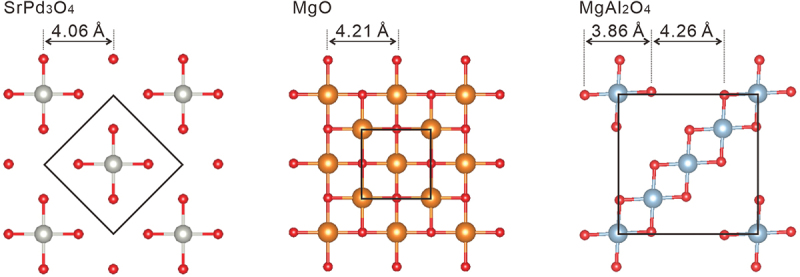
Surface atomic arrangements of SrPd_3_O_4_, MgO, and MgAl_2_O_4_. The black squares indicate the unit cells projected along the c-axis.

The failure of the epitaxial growth of SrPd_3_O_4_ on MgAl_2_O_4_ may be understood by the difference between the atomic arrangements of MgO and MgAl_2_O_4_ shown in Figure 6 [33]. In the cases of SrPd_3_O_4_ and MgO, oxide ions are equally spaced with a lattice mismatch of 2.1%, whereas the arrangement of the oxide ions is distorted from the square lattice for MgAl_2_O_4_. We speculate that the three-dimensional PdO_4_ chains of the NaPt_3_O_4_-type lattice may not be flexible enough to accommodate the unevenly spaced atomic arrangement of the MgAl_2_O_4_ surface lattice, leading to the unstable formation of SrPd_3_O_4_. The stiffness of the NaPt_3_O_4_-type crystal structure has also been pointed out in Ref. [41] in connection with the absence of structural transitions even with the one-dimensional chains composed of Pd *dz*^2^ orbitals, where Peierls transition is expected. This situation is contrasted with perovskite or rutile structures composed of *M*O_6_ (*M*: transition metal) octahedral networks, where the freedom of the octahedral rotation gives the flexibility to adapt the lattice to an isostructural substrate [42,43] even with a relatively large lattice mismatch over ~3% [44]. Thus, given this thought, NaPt_3_O_4_-type materials may not exhibit such a high controllability of electronic structure by epitaxial strain, unlike perovskite materials.

## 5. Electrical transport

**Figure 7.**
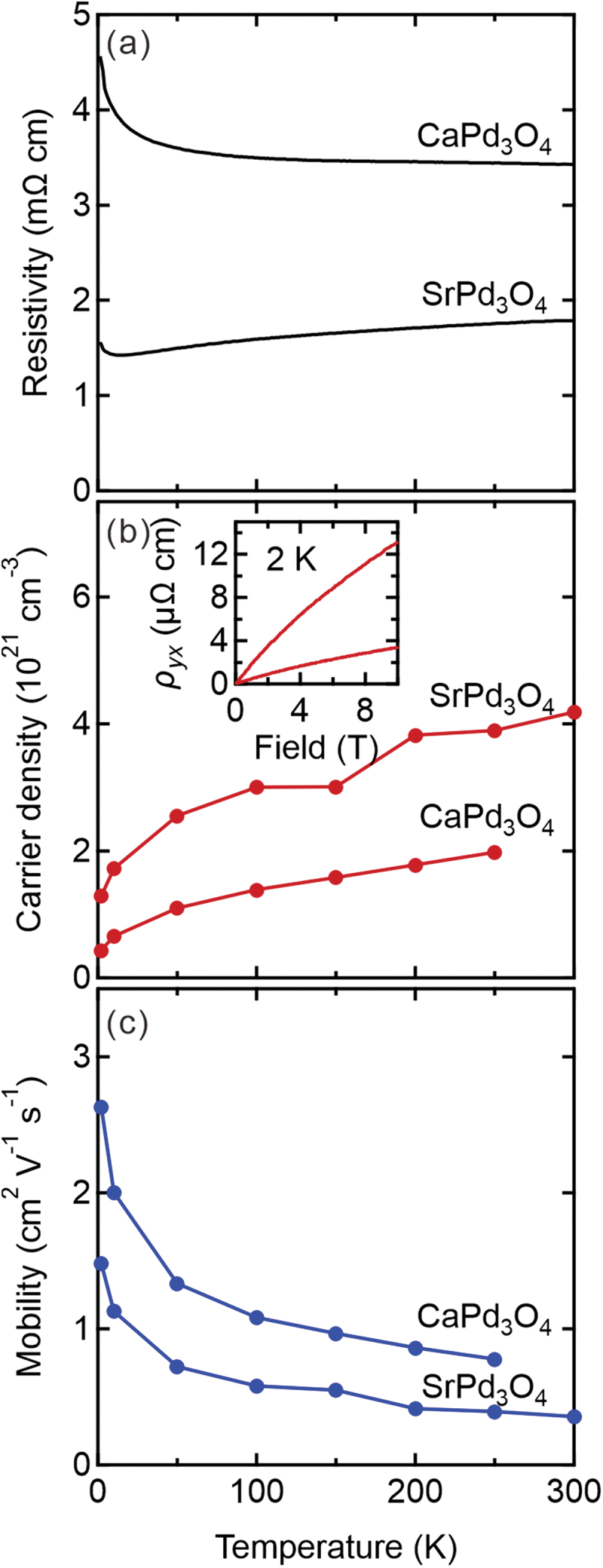
Temperature dependence of (a) resistivity, (b) carrier density, and (c) mobility of SrPd_3_O_4_, and CaPd_3_O_4_ thin films grown on MgO (100) substrate at 500°C under 0.2 Torr followed by annealing in air at 800°C. The carrier type measured from the Hall effect is *p*-type for both samples as shown in the inset of the panel (b).

We have measured the electrical transport of the SrPd_3_O_4_ and CaPd_3_O_4_ thin films as shown in Figure 7 for annealed samples. The CaPd_3_O_4_ thin film is weakly insulating, while SrPd_3_O_4_ thin film is metallic down to low temperature with a slight upturn below 10 K. Carrier density and mobility are also estimated from the Hall effect, which is in the order of 10^21^ cm^−3^ (hole-type) and 1 cm^2^ V^−1^ s^−1^, respectively, in both compounds. The carrier density of the thin films is about one order of magnitude larger than the previously reported values for bulk [23,24] probably due to non-stoichiometry introduced in the growth process. The temperature dependence data show that the carrier density decreases and the mobility gradually increases with decreasing temperature as typical behavior of a narrow-gap semiconductor. The mobility, although not specified in the previous reports, is comparable to the bulk value, if estimated from the resistivity and Hall coefficient.

## 6. Band calculations

To understand the electrical transport, we have carried out first-principles calculation. Since the band calculation with the generalized gradient approximation (GGA) for the exchange-correlation functional often underestimate the band gap of semiconductors, the previous theoretical study on CaPd_3_O_4_ employed modified Becke-Johnson (mBJ) meta-GGA functional [45] for the better estimation [46] and reported Dirac-type band crossings [20]. In this study, however, we have employed the HSE06 screened hybrid functional as a recent study shows that this functional qualitatively reproduces a small band gap observed for bulk CaPd_3_O_4_ [47].

**Figure 8.**
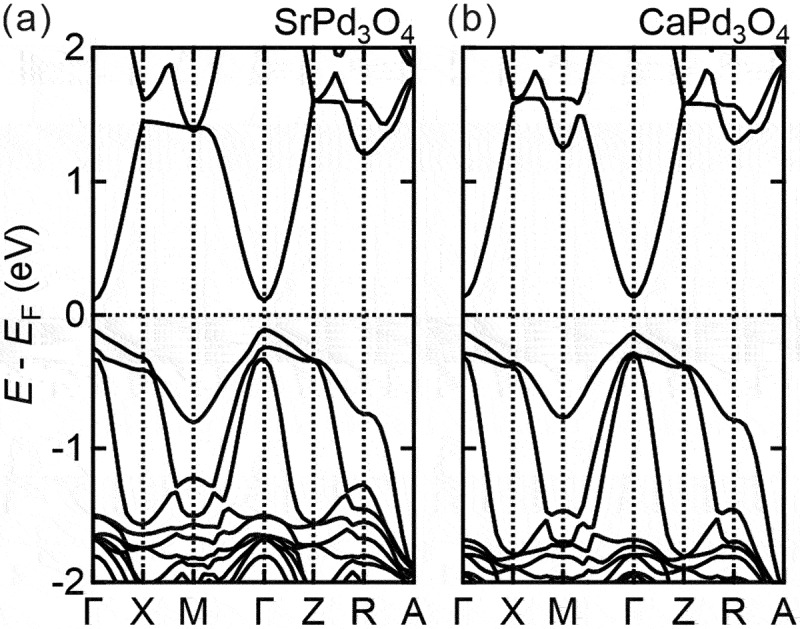
First-principles band calculations of (a) SrPd_3_O_4_ and (b) CaPd_3_O_4_ using lattice parameters extracted from the reciprocal space mapping. The calculated band gaps are 0.22 eV and 0.27 eV for SrPd_3_O_4_ and CaPd_3_O_4_, respectively.

Figure 8 shows the band calculation for SrPd_3_O_4_ and CaPd_3_O_4_ using lattice parameters estimated from the reciprocal space mapping. We find the band gaps open between the crystal field split bands between the *d*_*x*2-*y*2_ and *d*_*z*2_ orbitals. The values of the band gaps are 0.22 eV and 0.27 eV for SrPd_3_O_4_ and CaPd_3_O_4_, respectively [48]. We have also confirmed that the band structures in Figure 8 are not significantly different from those obtained using bulk lattice parameters [20] although the band gap of SrPd_3_O_4_ is slightly reduced by ~0.02 eV when the experimental lattice constants of the *A*Pd_3_O_4_ films grown on MgO substrate are used, compared with that calculated with the bulk lattice constant. This small variation of the band gap by strain indicates that it may be difficult to utilize epitaxial strain to drastically modulate the electronic structure in the case of NaPt_3_O_4_-type materials due to the inflexibility of the lattice in contrast to the case of perovskite materials, which can accommodate a large variation of strains by *M*O_6_ octahedral rotations as introduced above. Instead, the substitution of the *A*-site may work more effectively to control the band gap for *A*Pd_3_O_4_ through crystal field splitting between *d*_*x*2-*y*2_ and *d*_*z*2_.

In light of a recent experimental study, the value of the band gap for CaPd_3_O_4_ using HSE06 screened hybrid functional seems to be overestimated compared with that obtained from the optical spectroscopy, which is ~0.12 eV [47]. The band gap of SrPd_3_O_4_ is expected to be even smaller, close to band touching. Unfortunately, we cannot assess whether our films are Dirac semimetals, as suggested in Ref. [20] or narrow-gap semiconductors as in the bulk based on the transport data in Figure 7 due to a large number of extrinsic carriers. We have also tried to estimate the band gaps of our CaPd_3_O_4_ and SrPd_3_O_4_ films by optical spectroscopy but failed due to residual carriers causing large Burstein-Moss shifts and Drude peaks, which awaits the improvements of the film quality in the future.

## 7. Conclusion

In this study, we report the fabrication of SrPd_3_O_4_ and CaPd_3_O_4_ thin films by pulsed laser deposition together with structural and transport properties. X-ray diffraction and transmission electron microscopy have revealed that (100)-oriented thin films are grown on MgO (100) substrates under limited growth conditions following the thermodynamic-phase diagram of Pd oxides, whereas other substrates including SrTiO_3_, GdScO_3_, MgAl_2_O_4_ do not lead to clear crystallization within our investigation probably due to the inflexibility of the NaPt_3_O_4_-type crystal structure. The transport properties show a weak temperature dependence, suggestive of a narrow-gap semiconductor. However, a large number of extrinsic carriers are present in our films, which hinders us from accessing the properties originating from the possible topological bands. The first-principles band calculations show that these compounds may be narrow-gap semiconductors close to the topological-phase transition. The thin-film fabrication reported here will open a way to modulate the electronic structure by, for example, chemical substitution or pressure, which leads to a topological band structure.
